# Things Every Doctor Should Know

**Published:** 1897-01

**Authors:** 


### Things Every Doctor Should Know.

"In Medicine as a Profession," (*Boston Medical and Surgical Journal*), Dr. David W. Cheever quotes a number of authorities and decisions, which, alone, constitute quite a fund of knowledge, and make most useful and interesting reading. We have here abstracted them for the benefit of our readers, regretting that we cannot reproduce the entire article:

The oath of Hippocrates also forbade the taking of fetal life. It is the doctor's business to save life, not to destroy it. No occasion should tempt the physician to destroy an infant except to save the mother. Whether, under proper restrictions, he might be allowed to hasten the exit of the really moribund sufferer, and promote euthanasy, is a question.

*Greenleaf on Evidence* (Edition of 1896, vol. i, p. 148, under "Privileged Communications").—"Neither is protection extended to medical persons in regard to information they have acquired confidentially, by attending in their professional characters."

Protection by common law is not extended to priests; although the Roman Church regards the confessions as made to God, and forbids its revelation; and in the English Church, the clergyman telling is "under pain of irregularity." Anything told to legal counsel, however, is a privileged communication, and cannot be questioned, unless the party making it waives his right as to secrecy. Massachusettes courts go by the common law, and oblige the physician to tell the secret confidences of his patient, and to lay bare his diseases or his sins. Not so New

York, Missouri, Indiana, Nebraska, California, Wisconsin, Michigan, Minnesota—other States I have no knowledge of.

*Revised Statutes of New York* (vol. ii, p. 406, paragraphs 72, 73.—"No person duly authorized to practice medicine or surgery shall be allowed to disclose information acquired in attending a patient in a professional character, and which information was necessary to enable him to prescribe for him, or to do any act as a surgeon."

The New York statute has been held to apply also to knowledge acquired by observations of the patient's symptoms, or even the statements of others present. Paragraph 72 extends the same privilege to priests and ministers.

The doctor is not obliged to go when called; the only law which binds him is the obligation of humanity. Thus, if in an isolated community and no other physician were to be found, he might feel obliged, while he would not in a city or town where there were other doctors. Fatigue, sickness, overwork, other engagements, are sufficient excuses. Were it otherwise, he would soon die of exhaustion. He may decline, day or night; he can select his patients as he chooses; but if he goes, he feels obliged to continue.

*McClelland on Civil Malpractice* (p. 15).—"Among practitioners of the different schools, consultations cannot be held, for the reason that there is a radical difference between them either as to the medicines to be used, or the manner of using them; hence, if the practitioners be honest in their several beliefs, no good can accrue to the patient, this being the sole object."

Exorbant fees injure the standing of the profession. Liberal fees, in consultation, are a protection to the attending physician. Small fees to the poor—but some fee—is the true course for the young doctor.

"The law requires of a man who offers his services in any profession, three things: that reasonable degree of learning, skill and experience ordinarily possessed by others of his profession; reasonable and ordinary care in the treatment of the case committed to him; and the exercise of his best judgment in cases of doubt. . . . A physician does not engage to warrant and effect a perfect cure. . . . When the jury is satisfied of reasonable skill and care, that is sufficient."—*Sargent, J., Verdict for Defendant, McClelland*, pp. 32, 33.

"What constitutes *ordinary* or *reasonable* care or skill, and what is the proof of it? It is not easy to say. As much cannot be expected of physicians in remote localities, where they are cut off from opportunities of improvement, as from a physician living in a community where opportunity is afforded of seeing disease and accidents under more varied forms; nor from this class as from physicians connected with hospitals, or who reside in large cities. If it were otherwise, we should find but few physicians except in populous communities. The very favorable rule has been laid down in the law, that, the least amount of skill, therefore, with which a fair proportion of the practitioners of a given locality are endowed, is taken as the criterion by which to judge the physicians ability or skill."—*Bouvier's Inst.*, §§ 1004, 1005, *McClelland*, pp. 18, 19.

"He is not responsible in damages for want of success, unless it is shown to result from a want of ordinary skill and learning, and such as is ordinarily possessed by others of his profession, or from want of ordinary care or attention. He is not presumed to engage for extraordinary skill nor for extraordinary care; nor can he be made responsible in damage for errors in judgment, or mere mistakes in matters of doubt or uncertainty."—*See J. Foster*, 460 (28 Maine, 97; 39 Maine, 155); *McClelland*, p. 43.

"The defendant is not liable for want of the highest degree of skill, but for ordinary skill."—*Seare v. Prentice*, 8 East, 348; *Chitty on Contracts*, 165.

"And, of course, only for the want of ordinary care and ordinary judgment. The practice of surgery is indispensable to the community; and while damages should be paid for negligence and carelessness, surgeons should not be deterred from the pursuit of their profession by intemperate and extravagant verdicts. The compensation of surgeons in the country is small in comparison with wnat is paid in cities for similar services; and an error of judgment is visited with a severe penalty, which takes from one a larger share of the surplus earnings of life."—*Wells, J.*, *McClelland*, p. 260.

In England, a Medical Defence Union is in vigorous existence. It assumes the risks of suits for malpractice, and defends them. It is a mutual insurance company of medical men. Here, an attempt was made in 1895 to secure the assent of the Massachusetts legislature to the establishment of a medical and surgical branch of the Employers' Liability Insurance Company. It failed, however. It is the opinion of some lawyers that the existence of such an association to defend the doctor, would turn the jury against him.

